# Supplementary material for: Insight into the kinematics of blue whale surface foraging through drone observations and prey data
Source: PeerJ. 2020 Apr 22;8:e8906. doi: 10.7717/peerj.8906 (PMC7183305; doi:10.7717/peerj.8906)
Supplement: Table S1 [file peerj-08-8906-s001.docx]

**Supplementary Information**

Insight into the kinematics of blue whale surface foraging through drone observations and prey data

Leigh G. Torres, Dawn R. Barlow, Todd E. Chandler, Jonathan D. Burnett

**Table S1**. Mean and standard deviation of measured features for all krill aggregations (n = 2,911).

|  | **Mean** | **sd** |
| --- | --- | --- |
| Aggregation mean Sv | -82.17 | 9.17 |
| Aggregation mean depth | 44.36 | 30.91 |
| Aggregation thickness | 5.67 | 10.06 |
