# Supplementary material for: Insight into the kinematics of blue whale surface foraging through drone observations and prey data
Source: PeerJ. 2020 Apr 22;8:e8906. doi: 10.7717/peerj.8906 (PMC7183305; doi:10.7717/peerj.8906)
Supplement: Table S2 — F-test conducted with a 2-way mixed-effects model. 1Scores of groups of observations are correlated in an additive manner among raters. 2Different raters assign the same score to the same observation. Koo TK, Li MY (2016) A Guideline of Selecting and Reporting Intraclass Correlation Coefficients for Reliability Research. Journal of Chiropractic Medicine 15:155-163 [file peerj-08-8906-s002.docx]

**Supplementary Information**

Insight into the kinematics of blue whale surface foraging through drone observations and prey data

Leigh G. Torres, Dawn R. Barlow, Todd E. Chandler, Jonathan D. Burnett

**Table S2.** Results of the Interrater Correlation Coefficient (ICC; Koo & Li 2016) of consistency and agreement between the four independent co-author evaluations of the blue whale’s head inclination and roll estimates of 0.5 sec interval images. F-test conducted with a 2-way mixed-effects model.

|  |  | Interclass Correlation | | |  |  | F Test with True value = 0 |
| --- | --- | --- | --- | --- | --- | --- | --- |
| Observation | Measurement | Lower bound | Upper bound | Value | df1 | df2 | p |
| Head inclination | Consistency^1^ | 0.84 | 0.89 | 0.93 | 63 | 189 | < 0.001 |
| Roll | Consistency^1^ | 0.99 | 0.99 | 1 | 63 | 189 | < 0.001 |
| Head inclination | Aggreement^2^ | 0.66 | 0.91 | 0.83 | 63 | 189 | < 0.001 |
| Roll | Aggreement^2^ | 0.99 | 0.99 | 1 | 63 | 189 | < 0.001 |

^1^Scores of groups of observations are correlated in an additive manner among raters.

^2^Different raters assign the same score to the same observation.

Koo TK, Li MY (2016) A Guideline of Selecting and Reporting Intraclass Correlation Coefficients for Reliability Research. Journal of Chiropractic Medicine 15:155-163
