# Supplementary figures and images for: Insight into the kinematics of blue whale surface foraging through drone observations and prey data
Source: PeerJ. 2020 Apr 22;8:e8906. doi: 10.7717/peerj.8906 (PMC7183305; doi:10.7717/peerj.8906)

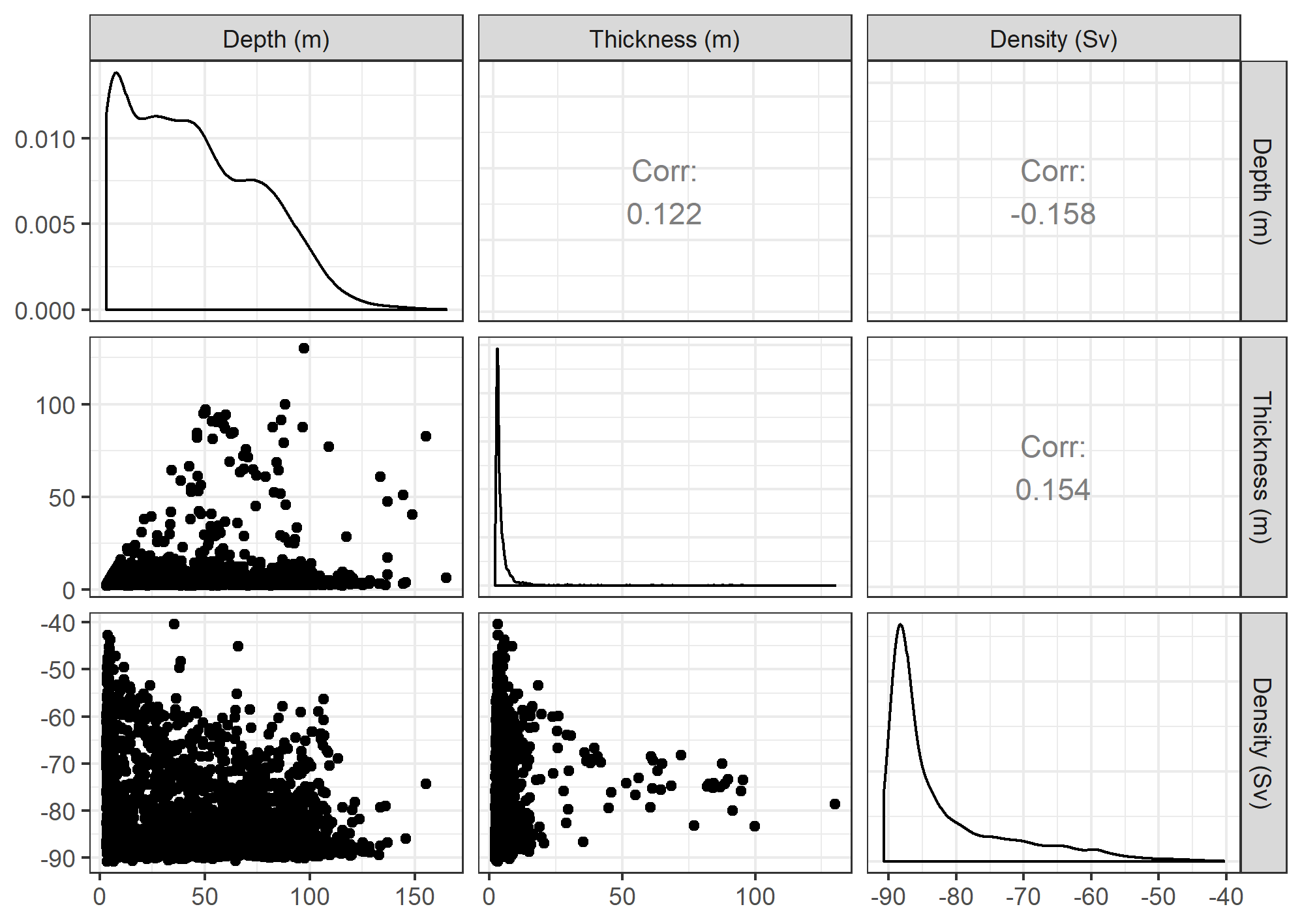

Supplement: Figure S1 — Plots on the diagonal show the distribution (probability density) of each metric. Plots in the upper right list the Pearson’s correlation coefficient between each pair, and scatter plots in the lower left show the relationship between each pair. [file peerj-08-8906-s003.png]

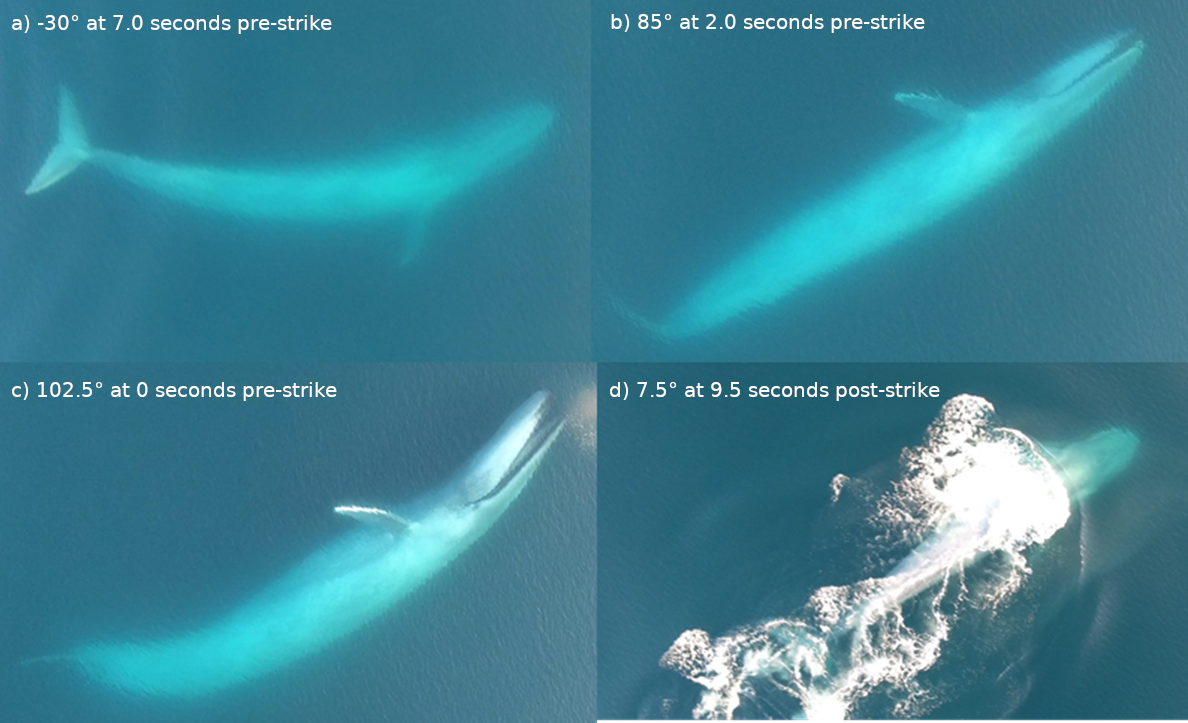

Supplement: Figure S2 — Each 0.5 sec image was evaluated by the 4 co-authors and the mean value is given with each image and time stamp. [file peerj-08-8906-s004.png]
