# Supplementary material for: Insight into the kinematics of blue whale surface foraging through drone observations and prey data
Source: PeerJ. 2020 Apr 22;8:e8906. doi: 10.7717/peerj.8906 (PMC7183305; doi:10.7717/peerj.8906)
Supplement: Supplemental Information 5 [file peerj-08-8906-s005.docx]

**Supplementary Information**

Insight into the kinematics of blue whale surface foraging through drone observations and prey data

Leigh G. Torres, Dawn R. Barlow, Todd E. Chandler, Jonathan D. Burnett

**Data Availability**

Data are made available in the following Figshare repository: <https://figshare.com/projects/Insight_into_the_significance_and_kinematics_of_blue_whale_surface_foraging_through_drone_observations_and_prey_data_Supplementary_Information_/74127>

The full Unmanned Aerial Systems (UAS) videos of the blue whale surface lunge feeding event and other surface events analyzed in this study can be viewed in the Figshare repository at the following link. Please refer to the time stamps in Table 1 of the manuscript to view the four foraging events described. The footage was filmed by Todd Chandler, and ownership of UAS videos belongs to Leigh Torres, Oregon State University. These UAS videos should only be used for scientific purposes and should not be shared on social media or broadly without explicit permission from L. Torres.

[10.6084/m9.figshare.11595246](https://doi.org/10.6084/m9.figshare.11595246)

Krill aggregation data files examined in this study (.csv files) and the Matlab script used to identify aggregations and their attributes is provided in the Figshare repository at the following link. Additionally, data on krill aggregation characteristics at blue whale sightings and at absence locations, and the R script used to analyze and plot these data, are also provided.

[10.6084/m9.figshare.11595261](https://doi.org/10.6084/m9.figshare.11595261)
